# Coxsackievirus B Infections Are Associated With the Risk of Islet Autoimmunity in Children With Strong Genetic Susceptibility to Type 1 Diabetes—Results From the TRIGR Divia Study

**DOI:** 10.1002/dmrr.70207

**Published:** 2026-07-29

**Authors:** Maarit Oikarinen, Amirbabak Sioofy‐Khojine, Leena Puustinen, David Cuthbertson, Jussi Lehtonen, Niila V. V. Jouppila, Johnny Ludvigsson, Leena Hakola, Sari Niinistö, Iris Erlund, Mikael Knip, Jeffrey P. Krischer, Suvi M. Virtanen, Heikki Hyöty, the TRIGR Investigators

**Affiliations:** ^1^ Faculty of Medicine and Health Technology Tampere University Tampere Finland; ^2^ Health Informatics Institute Morsani College of Medicine University of South Florida Tampa Florida USA; ^3^ Department of Biomedical and Clinical Sciences Division of Pediatrics, Crown Princess Victoria Children's Hospital Linköping University Linköping Sweden; ^4^ Faculty of Social Sciences Unit of Health Sciences Tampere University Tampere Finland; ^5^ Tampere University Hospital, Wellbeing Services County of Pirkanmaa Tampere Finland; ^6^ Department of Public Health Finnish Institute for Health and Welfare Helsinki Finland; ^7^ Institute for Nutrition and Health Research Helsinki Finland; ^8^ Research Program for Clinical and Molecular Metabolism Faculty of Medicine University of Helsinki Helsinki Finland; ^9^ Department of Pediatrics Tampere University Hospital Tampere Finland; ^10^ Center for Child Health Research Tampere University Tampere University Hospital Tampere Finland; ^11^ Fimlab Laboratories Pirkanmaa Hospital District Tampere Finland

**Keywords:** coxsackievirus, coxsackievirus B, enterovirus, IgG glass antibody, islet autoimmunity, microbial infection, neutralising antibody, serology, type 1 diabetes, viral infection

## Abstract

**Aims:**

Especially, the coxsackievirus B group of enteroviruses has been linked to the development of islet autoimmunity and type 1 diabetes in genetically susceptible individuals. Our aim was to study the possible associations of 10 different microbial infections with islet autoimmunity in a large international prospective study.

**Materials and Methods:**

In a nested case‐control study within the TRIGR study, follow‐up serum samples from 240 islet autoantibody‐positive case children and 436 age‐ and country‐matched control children were analysed for IgG class antibodies against 10 different respiratory and gastrointestinal microbes using an enzyme immunoassay, and for neutralising antibodies against all six coxsackievirus B types. The samples were obtained at several time points prior to and at the time of seroconversion for multiple islet autoantibodies. All children had a first‐degree relative with type 1 diabetes, and they carried risk‐associated HLA‐DQ alleles.

**Results:**

Coxsackievirus B5 was associated with an increased risk of islet autoimmunity (OR 2.22, 95% CI 1.29–3.80, *p* = 0.004, corrected *p* = 0.024), which remained after adjustment for HLA, sex, and maternal type 1 diabetes. This association was particularly seen for infections occurring more than 12 months (OR 2.02, 95% CI 1.06–3.84, *p* = 0.032) and 0–6 months (OR 2.66, 95% CI 1.11–6.35, *p* = 0.028) before the first detection of multiple islet autoantibodies. After correction for multiple comparisons, none of the other viruses showed an association with islet autoimmunity.

**Conclusions:**

This study supports previous evidence of the risk association of coxsackievirus B infections. These results support the role of certain virus infections as possible modulating factors in the pathogenesis of type 1 diabetes.

## 1 Introduction

Type 1 diabetes is caused by the selective destruction of insulin‐producing beta cells in the pancreas. The risk of developing type 1 diabetes is regulated by several, mostly HLA‐DQ, genes, and the beta cell damaging process has a clear autoimmune component. The increasing incidence of type 1 diabetes suggests that non‐genetic exposomic factors play an important role in the disease process and among them, viral infections have long been connected to the disease. Respiratory infections have shown an association with the initiation of the autoimmune process in a series of prospective studies [1, 2, 3, 4, 5]. In addition, certain specific viruses, particularly one subgroup of enteroviruses, coxsackievirus B (CVB) group, have been linked to the initiation of the process in several studies [6, 7, 8, 9]. Enterovirus infections usually manifest with respiratory symptoms, but several other viruses may also contribute to the observed association between respiratory infections and islet autoimmunity. In addition, some studies have suggested a risk association between gastroenteritis viruses, particularly rotavirus, and type 1 diabetes [10, 11, 12, 13].

However, few large prospective studies have been carried out to detect specific viral infections using laboratory assays in children who develop islet autoimmunity and progress to type 1 diabetes. Therefore, it would be important to carry out prospective studies to reliably identify viral associations, including those which have not been thoroughly addressed so far, such as viruses causing respiratory infections.

The present study aimed to assess the association of 10 microbial infections, including both respiratory and gastrointestinal infections, with the development of islet autoimmunity among children with a strong genetic risk for type 1 diabetes. Enteroviruses, coxsackievirus B group in particular, are among these microbes.

## 2 Materials and Methods

### 2.1 Study Material

The study comprised children recruited into the TRIGR (Trial to Reduce IDDM in the Genetically at Risk) study [14]. TRIGR is a double‐blind randomized clinical trial (ClinicalTrials.gov registration no. NCT00179777) of 2159 infants recruited between 2002 and 2007 in 15 countries (Australia, Canada, Czech Republic, Estonia, Finland, Germany, Hungary, Italy, Luxembourg, Netherlands, Poland, Spain, Sweden, Switzerland, USA). The inclusion criteria were having at least one first‐degree relative with type 1 diabetes, a risk HLA genotype for type 1 diabetes, and parental consent. Exclusion criteria have been described before [14]. All participants were observed until the youngest child turned 10‐year‐old in 2017.

Within the TRIGR cohort, altogether 2561 serum samples from 676 children, including 240 case children and 436 age‐matched (matched for both date of birth and age at the sample collection) and country‐matched control children, were included in the current nested case‐control study called Divia, which aims to study dietary and viral factors associated with islet autoimmunity. Sample sets were harmonised between the case and control children to have the same age points from both the case and the pair‐wise matched control children. The case children have been tested positive for at least two of the diabetes‐associated autoantibodies analysed, that is islet cell autoantibodies (ICA), GAD antibodies (GADA), insulin autoantibodies (IAA), and islet antigen 2 antibodies (IA‐2A). The control children tested negative for all autoantibodies by the time when the case child turned positive for multiple autoantibodies. Later, 183 of them developed positivity for one autoantibody. Of these 183, 122 were ICA‐positive, 36 were GADA‐positive, 24 were IAA‐positive, and one was IA‐2A‐positive. ICA were detected with the use of indirect immunofluorescence, while the three biochemical autoantibodies were quantified with specific radiobinding assays [15]. All cases with available controls were included in the study. The samples were analysed blinded for the case‐control status of the children. The method used to obtain information on sex: self‐reported. Characteristics of the study participants are described in Table 1.

**TABLE 1:** Characteristics of study subjects.

	Cases (*N* = 240)	Controls (*N* = 436)
Age at seroconversion for multiple autoantibodies in years, median (min‐max)	3.1 (0.5–12.3)	NA
Females, %	39.2	49.8
Region, %		
Northern Europe	24.6	25.2
Central and Southern Europe	28.3	28.0
North America	43.3	43.1
Australia	3.8	3.7
HLA risk, %^a^		
High risk	37.5	23.2
Moderate risk	38.8	44.5
Mild risk	23.8	32.3
Mother with type 1 diabetes, %	40.4	50.7

^a^ High risk: HLA‐DQB1*0302/DQB1*02; Moderate risk: HLA‐DQB1*0302/x (x not DQB1*02, DQB1*0301, or DQB1*0602); Mild risk: HLA‐DQA1*05‐DQB1*02/y (y not DQA1*0201‐DQB1*02, DQB1*0301, DQB1*0602, or DQB1*0603) and HLA‐DQA1*03‐DQB1*02/y (y not DQA1*0201‐DQB1*02, DQB1*0301, DQB1*0602, or DQB1*0603).

### 2.2 Virus Antibody Analyses

We analysed serum samples taken at the time of seroconversion for multiple diabetes‐associated autoantibodies and at several time points prior to that, that is, 6 months, 12 months, and 18 months preceding the seroconversion. These serum samples were analysed for the presence of IgG class antibodies against adenovirus, enterovirus, cytomegalovirus, influenza A virus, *mycoplasma*, norovirus, parainfluenza virus, respiratory syncytial virus, rhinovirus, and rotavirus at all the above‐mentioned time points using EIA, as previously described [16, 17]. In addition, the serum samples were tested for serotype‐specific neutralising antibodies against all six CVB serotypes (CVB1‐6), as previously described [8], at all the time points except for 18 months prior to the seroconversion (a sample taken 18 months before seroconversion was not available for neutralising antibody assay). The methods used in the current study have been described in detail in our previous publication [18]. However, we used a slightly different version of neutralising antibody assay compared to that used in our previous studies (virus‐serum mixture was incubated only for 45 minutes at +37°C and had no following overnight incubation step at +4°C). This modification mainly detects high affinity CVB antibodies, making the assay highly specific.

**FIGURE 1:**
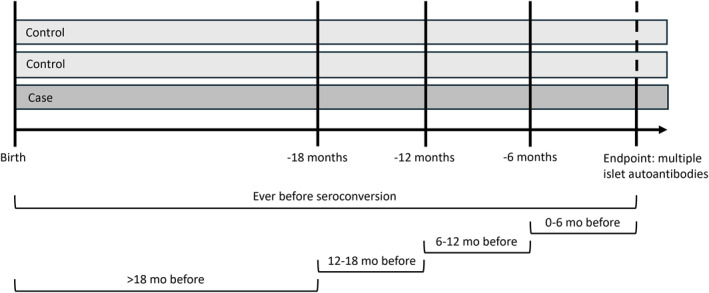
Study design. Serum samples from the case and control children were analysed for virus antibodies at different time points preceding the appearance of multiple islet autoantibodies. A sample taken 18 months before seroconversion was only available for EIA.

Possible microbial infections were assessed in each sample. In EIA, a two‐fold rise in the absorbance value of two consecutive samples was considered to indicate an infection between those two time points (e.g., if a two‐fold rise was observed in a sample taken 6 months prior to the seroconversion, infection occurred between the time points of 12 and 6 months prior to the seroconversion). Nonetheless, only an absorbance value of 0.4 or higher was considered to mark an infection. In neutralising antibody assays, seroconversion to antibody positivity, once antibodies remained positive in all later follow‐up samples tested, was considered to indicate an infection between the last negative and first positive time point. Disappearance kinetics of maternal antibodies (measured from cord blood) were taken into account when diagnosing infections in children before the age of 1 year. The study design is described in Figure 1.

### 2.3 Statistics

To study the association between microbial infections and the risk of islet autoimmunity, conditional logistical regressions were applied. These models examined the prevalence of each infection between cases and controls by providing odd ratios and their confidence intervals adjusted for covariates. Separate models were generated for each sample collected: at seroconversion, 6 months prior to seroconversion, 12 months prior to seroconversion, 18 months prior to seroconversion as well as ever prior to seroconversion. Additionally, conditional logistic regressions were utilised to model the timing of when each infection occurred prior to seroconversion (i.e., at seroconversion, 0–6 months prior to seroconversion, 6–12 months prior to seroconversion, 12–18 months prior to seroconversion, > 18 months prior to seroconversion). All analyses were carried out both unadjusted and adjusted for HLA risk, sex, and maternal type 1 diabetes. Children with missing data were excluded from the respective analyses.

The observed *p* values were multiplied by the number of comparisons made in the primary analyses (infections at any time point prior to the appearance of multiple islet autoantibodies) using Bonferroni correction (10 comparisons in EIA and 6 comparisons in neutralising antibody analyses). *p* values < 0.05 were considered statistically significant. The statistical analyses were performed using SAS version 9.4.

### 2.4 Ethical Aspects

The study was conducted according to the standards for research ethics including human participants. All human biological samples were collected from patients whose legal guardian provided written informed consent and with the approval of the local ethics committee and in accordance with the Declaration of Helsinki.

## 3 Results

The infections were documented by detecting diagnostic increases in IgG levels against 10 different microbes between serial follow‐up samples in the EIA assay, and in neutralising antibodies against each of the six CVB types. The proportion of case and control children who had at least one infection before the case children developed multiple islet autoimmunity varied between microbes ranging from 3% to 63%, norovirus, influenza A virus and enterovirus infections being the most common ones (Figure S1).

### 3.1 Association of Microbial Infections With Islet Autoimmunity

When children who developed islet autoimmunity were compared to their matched control children, having at least one CVB5 infection during the follow‐up was associated with increased risk of islet autoimmunity (OR 2.22, 95% CI 1.29–3.80, *p* = 0.004, corrected *p* = 0.024; Table 2) and this association also remained when adjusted for HLA, sex, and maternal type 1 diabetes. None of the other viruses showed a statistically significant association with islet autoimmunity after correction for the number of comparisons. The same association for CVB5 was seen in countries excluding Finland (189 cases and 341 controls; OR 2.09, 95% CI 1.19–3.69, *p* = 0.011) and countries excluding Northern Europe (181 cases and 326 controls; OR 2.23, 95% CI 1.24–4.02, *p* = 0.008), whereas only in Finland (51 cases and 95 controls; OR 3.61, 95% CI 0.65–19.91, *p* = 0.141) and only in Northern Europe (59 cases and 110 controls, OR 2.16, 95% CI 0.57–8.14, *p* = 0.256) this association was not significant likely due to the smaller number of subjects. In addition, when only those children who progressed to clinical type 1 diabetes were included in the analyses, this association remained close to being significant (143 cases and 263 controls; OR 2.374, 95% CI 0.97–5.82, *p* = 0.059). Also, when excluding those control children who later developed positivity for single autoantibody and only including the control children who remain autoantibody‐negative, CVB5 infections, and not any other infections, during the follow‐up were associated with increased risk of autoimmunity (OR 2.331, 95% CI 1.30–4.20, *p* = 0.005).

**TABLE 2:** Proportion of children with microbial infections and risk of islet autoimmunity for multiple autoantibodies at any time point prior to the appearance of multiple islet autoantibodies (i.e., at the time of seroconversion, and 6 months, 12 months, and 18 months preceding the autoantibody seroconversion).

Antibody	Cases (*N* = 240)	Controls (*N* = 436)	Unadjusted				Adjusted^a^			
	Virus antibody positive (%)	Virus antibody positive (%)	OR	95% CI	*p*	*p* corr^b^	OR	95% CI	*p*	*p* corr^b^
Adenovirus	51	49	1.02	0.74–1.42	0.890		1.01	0.72–1.42	0.950	
Cytomegalovirus	15	14	1.18	0.75–1.87	0.474		1.19	0.74–1.93	0.475	
Enterovirus	54	58	0.81	0.57–1.14	0.228		0.83	0.57–1.18	0.296	
*Mycoplasma*	17	14	1.24	0.77–1.99	0.338		1.24	0.75–2.05	0.411	
Norovirus	58	65	0.64	0.45–0.93	**0.018**	0.180	0.64	0.44–0.94	**0.022**	0.220
Respiratory syncytial virus	52	55	0.80	0.56–1.14	0.207		0.85	0.59–1.22	0.371	
Influenza A virus	65	60	1.35	0.91–2.01	0.138		1.29	0.86–1.93	0.224	
Parainfluenza virus	32	24	1.56	1.04–2.33	**0.030**	0.300	1.70	1.12–2.60	**0.014**	0.140
Rhinovirus	49	45	1.22	0.87–1.71	0.250		1.24	0.87–1.77	0.236	
Rotavirus	37	34	1.12	0.78–1.62	0.533		1.22	0.83–1.80	0.305	
Any infection	95	97	0.45	0.18–1.14	0.091		0.43	0.16–1.14	0.089	

The neutralising antibody	Cases (*N* = 239)	Controls (*N* = 415)	Unadjusted				Adjusted^a^			
	Virus antibody positive (%)	Virus antibody positive (%)	OR	95% CI	*p*	*p* corr^b^	OR	95% CI	*p*	*p* corr^b^
CVB1	18	19	0.95	0.62–1.46	0.828		0.99	0.64–1.54	0.976	
CVB2	16	20	0.80	0.51–1.25	0.319		0.73	0.45–1.17	0.177	
CVB3	11	13	0.92	0.53–1.57	0.750		1.01	0.58–1.77	0.977	
CVB4	14	12	1.17	0.69–1.99	0.555		1.26	0.73–2.18	0.409	
CVB5	18	11	2.22	1.29–3.80	**0.004**	**0.024**	2.42	1.38–4.24	**0.002**	**0.012**
CVB6	4	2	1.72	0.65–4.57	0.275		1.86	0.69–5.03	0.222	
Any CVB infection	46	48	0.95	0.65–1.38	0.774		0.99	0.67–1.46	0.968	

*Note:* All the *p* values that are significant (i.e., *p* value is < 0.05) are marked in bold.

^a^ Adjusted for HLA, sex, and maternal type 1 diabetes.

^b^ Corrected for the number of comparisons (Bonferroni correction).

Prior to seroconversion, the case children were infected on average with 4.30 different viruses and with 0.81 different CVB types, and for the control children, the corresponding figures were 4.18 different viruses and 0.77 different CVB types.

### 3.2 Timing of Infections

Next, we analysed the timing of infections in relation to the appearance of multiple islet autoantibodies. The risk association between coxsackievirus B5 infections and islet autoimmunity was particularly seen in infections occurring more than 12 months prior to the first detection of multiple islet autoantibodies (OR 2.02, 95% CI 1.06–3.84, *p* = 0.032; Table 3) as well as 0–6 months before seroconversion (i.e., in the same time window as autoantibodies) (OR 2.66, 95% CI 1.11–6.35, *p* = 0.028; Table 3). These associations remained after adjustment for HLA, sex, and maternal type 1 diabetes. Instead, the association was not significant 6–12 months before seroconversion due to the smaller number of samples in this time window.

**TABLE 3:** The risk of islet autoimmunity for multiple autoantibodies associated with CVB5 infections according to the time when these infections were diagnosed in all children and in children whose first autoantibody‐positive sample was already positive for multiple autoantibodies.

		Unadjusted			Adjusted^a^		
		OR	95% CI	*p*	OR	95% CI	*p*
All children	Ever before the seroconversion	2.22	1.29–3.80	**0.004**	2.42	1.38–4.24	**0.002**
	0–6 months before seroconversion	2.66	1.11–6.35	**0.028**	3.21	1.28–8.04	**0.013**
	6–12 months before seroconversion	2.22	0.31–15.90	0.429	1.15	0.15–9.16	0.893
	> 12 months before seroconversion	2.02	1.06–3.84	**0.032**	2.26	1.16–4.40	**0.016**
Children with multiple aab in the first aab + sample	Ever before the seroconversion	3.31	1.22–8.96	0.019	3.28	1.14–9.46	0.028
	0–6 months before seroconversion	2.22	0.48–10.3	0.311	1.72	0.33–9.14	0.522
	6–12 months before seroconversion	0.00	0.00‐inf	0.991	0.00	0.00‐inf	0.992
	> 12 months before seroconversion	3.55	1.03–12.25	**0.045**	4.08	1.11–15.07	**0.035**

*Note:* Bold values stand for values that are statistically significant (*p* < 0.05).

^a^ Adjusted for HLA, sex, and maternal type 1 diabetes.

### 3.3 Sensitivity Analyses

To specifically address the time‐dependencies between infections and appearance of islet autoimmunity, we carried out sensitivity analyses among those children whose first autoantibody positive sample was already positive for multiple autoantibodies. These analyses included 100 case children and their 181 matched controls. In these children, the time when islet autoimmunity started can be accurately compared to the timing of preceding infections. Association of coxsackievirus B5 infections at any time point prior to the seroconversion to positivity for islet autoimmunity remained in the sensitivity analyses (Table 3). This association was statistically significant for infections that occurred more than 12 months prior to the first detection of islet autoantibodies (OR 3.55, 95% CI 1.03–12.25, *p* = 0.045; Table 3). This association also remained in the adjusted analyses.

### 3.4 Effect of Rotavirus Vaccination

Based on questionnaire data, rotavirus vaccinations were administered to 186 of the 676 children (27.5%) prior to when the case developed islet autoimmunity. Therefore, we carried out additional analyses by adjusting for rotavirus vaccinations. In these vaccination‐adjusted analyses, no association was seen between rotavirus infections and islet autoimmunity when all rotavirus infections prior to seroconversion were included in the analyses (OR 1.21, 95% CI 0.76–1.93, *p* = 0.427). We also repeated the analyses by excluding all rotavirus vaccinated children. Again, no association was seen in infections that occurred ever before autoantibody seroconversion (OR 1.28, 95% CI 0.69–2.37, *p* = 0.433).

## 4 Discussion

The current study supports the previously reported association between CVB infections and the increased risk of islet autoimmunity. This association was linked to CVB5 type, which has been associated with type 1 diabetes in previous studies as well [19, 20]. However, other CVB types showed no such association, which is in contrast to for example, the prospective Finnish DIPP study in which CVB1 was associated with a higher risk of islet autoimmunity [6]. Other studies have also linked CVB4 to islet autoimmunity [21]. Altogether, this suggests that any CVB type may have a diabetogenic potential, and different CVB types may turn out to be a risk virus in different populations and time periods, depending on the epidemiological situation and circulating CVB strains. Previous studies have also indicated that different CVB types may modulate each other's risk association possibly due to immunological cross‐protection between CVB types [6], which might also result in variation between the studies. The diabetogenic potential of CVBs could be linked to their tropism to the pancreatic islets, as described in autopsy studies [22, 23, 24, 25], and the intensive expression of coxsackievirus and adenovirus receptor (CAR) on beta cells [24, 26, 27].

Screening of other infections was carried out by measuring IgG class antibodies using EIA. While EIA is an excellent assay for the detection of antibodies against many viruses, the wide cross‐reactivity of EIA antibodies between different enterovirus types makes it unable to distinguish infections which are caused by a specific enterovirus type from infections caused by other enterovirus types (> 115 types altogether) [28, 29, 30, 31]. Since the CVB group of enteroviruses (6 types) has been linked to type 1 diabetes, we used neutralising antibody assay to detect type‐specific antibodies against each CVB. In fact, widely cross‐reactive antibodies may mask possible associations between the relatively small enterovirus group of CVBs and islet autoimmunity when the EIA assay is used. In line with this, a clear risk association was found between CVB5 and islet autoimmunity using a neutralising antibody assay, while the EIA assay did not show an association between enterovirus infections and islet autoimmunity.

The current study cohort included children who had a first‐degree relative affected by type 1 diabetes and disease‐associated HLA‐DQ alleles. Thus, their risk of developing type 1 diabetes was higher than that in most other prospective studies carried out so far. Therefore, the results of the current study provide an important addition to the existing literature by further supporting the association between CVB infections and islet autoimmunity in children with particularly strong genetic disease susceptibility. Another important aspect is that the study covered altogether 15 countries and included heterogenous populations. Thus, even though the epidemiology of CVBs differs according to time and place, their association with islet autoimmunity was still seen in this international study. In addition, the time‐relationship between CVB infections and islet autoimmunity is in line with previous observations suggesting varying time lag and a relatively slow operating mechanism in virus‐induced beta cell damage. Possible viral persistence has previously been implicated in enterovirus‐induced type 1 diabetes [32, 33, 34] and could be involved in such a long lag time in some children. It should be noted that the time when the case child turned positive for multiple islet autoantibodies was used as the study end point. Thus, part of the case children turned positive for a single autoantibody already prior to the study end point. Therefore, we carried out additional analyses among children who instantly developed multiple autoantibodies without prior detection of any of the tested autoantibodies. Thus, in these children, the exact time of the initiation of islet autoimmunity was known. These analyses showed that CVB5 was associated with the appearance of autoantibodies and showed a similar lag time of several months between the CVB infection and the initiation of islet autoimmunity. This finding suggests that CVB infections may contribute to the initiation of beta cell autoimmunity.

Rotavirus infections were not associated with islet autoimmunity. This is an important observation since only a few prospective studies have previously addressed the possible role of rotavirus in the initiation of islet autoimmunity [10]. The results are in line with our previous prospective study in the Finnish DIPP study cohort showing no association between rotavirus infections and islet autoimmunity [35]. The exclusion of children who had received rotavirus vaccination from these analyses as well as adjusting for rotavirus vaccinations did not change this result.

A recent prospective study showed that gastroenteritis may modulate the risk of islet autoimmunity, presenting either a risk or protective association depending on the timing of the infection [36]. These patterns were associated with the detection of norovirus in stool samples. Norovirus is a common cause of gastroenteritis in young children. However, in the present study, norovirus infections were not associated with islet autoimmunity (the inverse association that was seen in the primary analyses was not statistically significant after correction for multiple comparisons).

The study has some limitations to be considered when interpreting the results. Initiation of type 1 diabetes autoimmunity already begins with the appearance of a single diabetes‐associated autoantibody. A minority of the control children already had one autoantibody (most commonly ICA), which indicates that the process leading to type 1 diabetes may have already started in these children. However, the additional subgroup analyses showed that CVB5 infection was also associated with islet autoimmunity among such case‐control pairs in which the control child remained completely autoantibody negative. One additional aspect is that we used a modification of the neutralising antibody assay that mainly detects high‐affinity CVB antibodies. While this assay is highly specific, it fails to detect low‐affinity CVB antibodies. Therefore, it is possible that we missed a part of the CVB infections and underestimated their prevalence in this study population. One limitation of the study is that virus infections were detected only using serology and direct virus detection was not performed. For example, enterovirus detection requires stool or respiratory samples, but such samples were not collected in the TRIGR study. Therefore, part of the infections may have been missed in the present study.

In conclusion, this study is in line with the previously documented association of CVB infections with an increased risk of islet autoimmunity in children with genetic risk for type 1 diabetes. This finding supports the ongoing efforts to develop vaccines against coxsackie B viruses and test their efficacy in the prevention of type 1 diabetes [37].

## Author Contributions

M.O., A.S.K., L.P., D.C., J.Le, J.Lu., L.H., S.N., M.K., J.P.K., S.M.V., and H.H. were involved in the conception, design, and conduct of the study and the analysis and interpretation of the results. M.O., A.S.K., L.P., N.V.V.J., and H.H. contributed to the analyses of virus antibodies. J.Le. and D.C. were responsible for the statistical analyses. M.O. wrote the first draft of the manuscript, and all authors edited, reviewed, and approved the final version of the manuscript. M.O. is the guarantor of this work and, as such, had full access to all the data in the study and takes responsibility for the integrity of the data and the accuracy of the data analysis.

## Funding

This work was supported by the European Union's Horizon 2020 research and innovation programme under grant agreement No 874864 HEDIMED, Yrjö Jahnsson Foundation, Päivikki and Sakari Sohlberg Foundation, National Institutes of Health (grants 1DP3DK106918‐01, HD040364, HD042444, and HD051997), the Eunice Kennedy Shriver National Institute of Child Health and Development (NICHD), National Institute of Diabetes and Digestive and Kidney Diseases, Canadian Institutes of Health Research, JDRF, the Commission of the European Communities (specific RTD programme Quality of Life and Management of Living Resources, contract QLK1‐2002‐00372 Diabetes Prevention), the European Foundation for the Study of Diabetes/JDRF/Novo Nordisk Focused Research Grant, Academy of Finland (Centre of Excellence in Molecular Systems Immunology and Physiology Research 2012‐2017, Decision No. 250114, Decision No. 339922), Dutch Diabetes Research Foundation, Juho Vainio Foundation, Finnish Cultural Foundation, and Finnish Diabetes Research Foundation. The study sponsors were not involved in the design of the study, the collection, analysis, and interpretation of data, writing the report, or the decision to submit the report for publication.

## Conflicts of Interest

M.K. and H.H. are shareholders and members of the board of Vactech Ltd. which develops vaccines against picornaviruses. Other authors declare no conflicts of interest.

## Supporting information


Supporting Information S1

**Figure S1:** Proportion (%) of children (both cases and controls included) with microbial infections at any time point prior to the appearance of multiple islet autoantibodies.

## Data Availability

The datasets generated during and/or analysed in the current study are available from the corresponding author upon reasonable request.
