# Supplementary figures and images for: Coxsackievirus B Infections Are Associated With the Risk of Islet Autoimmunity in Children With Strong Genetic Susceptibility to Type 1 Diabetes—Results From the TRIGR Divia Study
Source: Diabetes Metab Res Rev. 2026 Jul 29;42(6):e70207. doi: 10.1002/dmrr.70207 (PMC13417744; doi:10.1002/dmrr.70207)

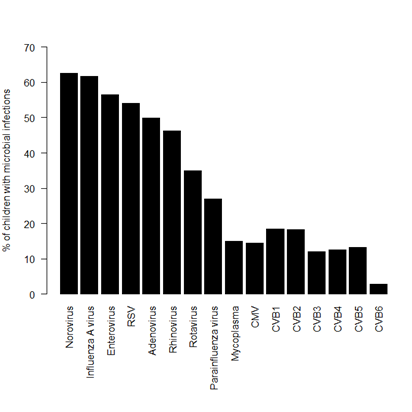

Supplement: Supplementary file 2 — Figure S1: Proportion (%) of children (both cases and controls included) with microbial infections at any time point prior to the appearance of multiple islet autoantibodies. [file DMRR-42-e70207-s002.tif]
